# How Thailand eliminated lymphatic filariasis as a public health problem

**DOI:** 10.1186/s40249-019-0549-1

**Published:** 2019-05-27

**Authors:** Sunsanee Rojanapanus, Tanaporn Toothong, Patcharida Boondej, Suwich Thammapalo, Naraporn Khuanyoung, Weena Santabutr, Preecha Prempree, Deyer Gopinath, Kapa D. Ramaiah

**Affiliations:** 1 Bureau of Vector Borne Diseases, Department of Disease Control, Ministry of Public Health, Nonthaburi, Thailand; 2 Office of Disease Prevention and Control, Ministry of Public Health, Songkhla, Thailand; 3 World Health Organization, Country Office for Thailand, Nonthaburi, Thailand; 4 Consultant on lymphatic filariasis, Tagore Nagar, Pondicherry, India

**Keywords:** Lymphatic filariasis, Elimination, Validation, Thailand

## Abstract

**Background:**

Lymphatic filariasis is endemic in nine of the eleven Member States of the World Health Organization South East Asia Region. This article describes the intensive interventions with the National Programme for Elimination of Lymphatic Filariasis in Thailand since its launch in 2001 till the validation of its elimination in 2017.

**Methods:**

A baseline epidemiological survey was initiated in 2001 to identify both brugian and bancroftian filarial areas and delineate its endemicity. Mass drug administration (MDA) with diethylcarbamazine citrate (DEC) and albendazole (ALB) was implemented in a total of 357 implementation units (IUs) in 11 lymphatic filariasis (LF) endemic provinces. The implementing unit (IU) was a sub-village. Stop-MDA surveys were conducted in 2006 in the 11 LF endemic provinces among population over 6 years of age and children of ≤6 years using immunochromatographic test (ICT) for *Wuchereria bancrofti* antigen and microfilariae (mf) detection for *Brugia malayi*. In Narathiwat province, Stop-MDA surveys were done in 2011 using ELISA. Transmission assessment surveys (TAS) were conducted in 2012–2013, 2015 and 2016–2017 among school students in the 6–7-year age-group. Surveillance of migrant populations through the national migrant health checkup were intensified in seven provinces over 2002–2017 for LF antigenaemia using ICT test cards. In four *B. malayi* endemic provinces, annual surveys to detect LF reservoir in domestic cats commenced in 1994. A 2001 survey of the chronic disease burden for LF established a register of the cumulative number of people with lymphedema/elephantiasis.

**Results:**

A total of five rounds of MDA annually were implemented over 2002–2006 in all IUs. Additional annual rounds of MDA were required in 87 IUs of Narathiwat province from 2007 to 2011 due to persistent infection. The annual national drug coverage with MDA over 2002–2012 was in the range of 68.0 to 95.4%. Stop-MDA surveys in 2006 in the 11 LF endemic provinces found nine mf positive cases in seven IUs in Narathiwat province with the highest prevalence of 0.8% (range: 0.1–0.8%). In Narathiwat TAS-1, TAS-2 and TAS-3 detected below transmission threshold rates for *B. malayi* mf among antibody positive children (0.3, 0.2 and 0.7% respectively). Contact tracing both all mf cases in all three TAS yielded no positive cases.

Through the migrant health checkup, a total of 23 477 persons were tested, showing a positive rate of 0.7% (range: 0.1–2.7%) over years 2002–2017. In Narathiwat province, annual ivermectin treatment among cats commenced in 2003 resulting in a decline of mf prevalence among cats from 8.0% in 1995 to 0.8% in 2015. As of April 2017, a total of 99 lymphoedema/elephantiasis patients were registered and followed-up under 34 health facilities.

**Conclusions:**

Thailand over the years 2002 to 2011 conducted extensive MDA with high coverage rates. Through periodic and regular monitoring surveys it delineated LF transmission areas at sub-village level and demonstrated through its evaluation surveys – the Stop-MDA surveys and TAS, below transmission threshold rates that enabled its validation of LF elimination. In September 2017, World Health Organization acknowledged the Ministry of Health Thailand had eliminated lymphatic filariasis as a public health problem.

**Electronic supplementary material:**

The online version of this article (10.1186/s40249-019-0549-1) contains supplementary material, which is available to authorized users.

## Multilingual abstracts

Please see Additional file 1 for translations of the abstract into the five official working languages of the United Nations.

## Background

### Eepidemiological status of lymphatic filariasis

Historically, lymphatic filariasis (LF) had been endemic only in some parts of Thailand, with both brugian and bancroftian filariasis being reported [1–5]. The first survey for LF was recorded as early as 1949 by the Department of Health, Ministry of Public Health (MoPH) and found that there was lymphatic filariasis, lymphoedema cases in six southern provinces; Chumphon, Surat Thani, Nakhon Si Thammarat, Phatthalung, Pattani and Narathiwat. Between 1951 and 1952 the World Health Organization (WHO) conducted LF blood surveys in four provinces of Surat Thani, Nakhon Si Thammarat, Phatthalung and Pattani, and found microfilarial positive rate averaging 21.0% (2.9–40.8%), all cases were *Brugia malayi* infection. The elephantiasis rate was 5.2%. The vector identified were *Mansonia* spp. (four species) and *Anopheles* spp. (five species) infected with infective stage larvae of *B. malayi*. The disease was recognized as being of public health importance in 1953. Between 1960 and 1961 Faculty of Tropical Medicine, Mahidol University and the Department of Health conducted LF surveys in three districts of Nakhon Si Thammarat province and detected 1246 cases. Between 1961 and 1988 numerous LF surveys were conducted in provinces of Surat Thani, Chumphon, Kanchanaburi and Mae Hong Son. The microfilariae (mf) surveys were then expanded over 1994–1995 to cover 32 provinces. In highly endemic provinces of Narathiwat > 900 village-surveys (includes first ever survey of hundreds of villages and resurvey of some villages to evaluate the impact of diethylcarbamazine citrate [DEC] treatment) were done during 1978–2001 and in Tak province > 600 village-surveys were performed over 1986–2001. In some provinces, notably Phang Nga where prevalence was confined to only a few villages, repeated treatment with DEC enabled the eventual elimination of LF.

### Programme structure in Thailand

**Fig. 1:**
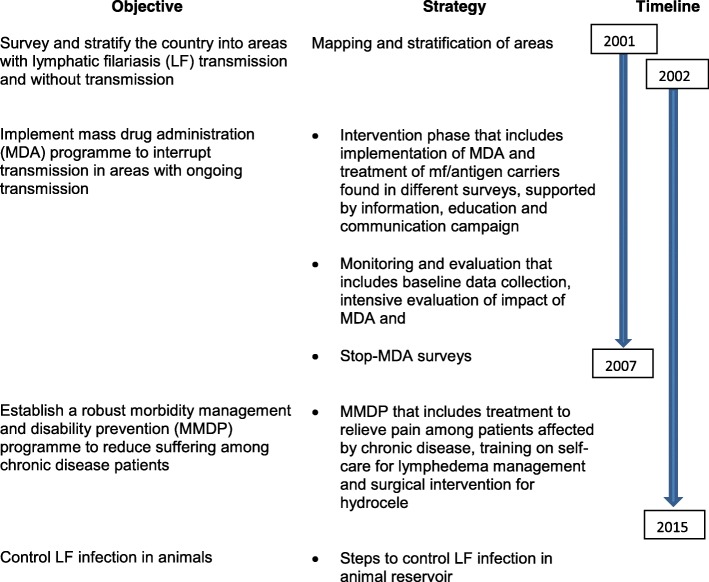
National Programme for Elimination of Lymphatic Filariasis in Thailand: Strategies, objectives and timelines

**Fig. 2:**
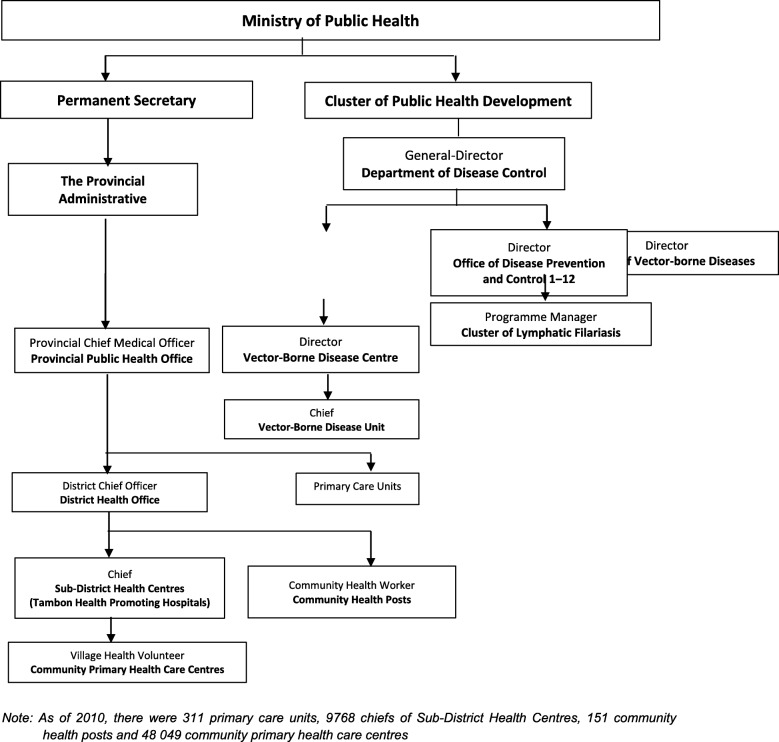
Structure of National Programme for Elimination of Lymphatic Filariasis in Thailand

In the year 1961, the Division of Lymphatic Filariasis was established under the Department of Health, MoPH with a primary strategy of using DEC to control LF in known endemic areas [6]. The MoPH of Thailand launched the National Programme to Eliminate LF (NPELF) in Thailand in 2001. The NPELF strategies and its objectives are shown in Fig. 1 and initially aimed to cease mass drug administration (MDA) by 2007 [7, 8]. The structure and organization of the programme is shown in Fig. 2. The Bureau of Vector-Borne Diseases (BVBD) under the Department of Disease Control (DDC) in the MoPH implements vector control and disease control programmes. Within the BVBD, the Cluster of Lymphatic Filariasis, headed by a programme manager coordinates the implementation of the NPELF.

At the province level, the respective Provincial Health Office (PHO), headed by the provincial chief medical officer coordinates the implementation of the programme, particularly the MDA activities, within the endemic provinces through liaising with DHOs. The provincial Vector Borne Disease Centre (VBDC) plays an important role in the monitoring and evaluation (M&E) and surveillance activities. The District Health Office (DHO) interacts with sub-district and village level health workers and monitors the implementation of the programme, actively supported by the district level Vector Borne Disease Unit (VBDU) in M&E and surveillance activities. The staff of the Primary Care Units (PCU), Sub-district Health Centres (SDHC), Community Health Posts (CHPs) and Community Primary Health Care Centres (CPHCC) implement various activities of the programme such as MDA, M & E and surveillance and Morbidity Management and Disability Prevention (MMDP).

### Delineation of endemicity

As a result of control measures and socio-economic improvement, LF distribution become very focal and restricted to some sub-villages. In the year 2001, when preparations started for establishing the NPELF, all historical data on prevalence up to sub-village level including environmental conditions of all the provinces were carefully examined and 11 provinces were declared endemic for LF.

### MDA as the major intervention for the LF elimination programme

The MDA programme was launched in the year 2002 and was implemented annually in consecutive years from 2002 to 2006 in endemic implemented units (IUs) of 11 provinces involving a total of 357 IUs with a total population of 124 496 (year 2002). As recommended by the WHO, DEC and Albendazole (ALB), were used in the MDA programme [8]. The dosage used was DEC at 6 mg/kg body weight plus a fixed dose of 400 mg ALB for each individual.

The quantities of medicines required for MDA for each IU was estimated annually. DEC tablets were procured from the local pharmaceutical companies by the MoPH and ALB received as donation from the donor pharmaceutical company, GlaxoSmithKline (GSK), through WHO South-east Asia Regional Office (SEARO). DEC tablet formulations included 50 mg and 300 mg, and ALB 400 mg. The quality of local DEC was assessed per the MoPH Food and Drug Administration (FDA) guidelines and found to meet the standards. Medicines were always procured at least 2–3 months in advance of the MDA activity to avoid delays. The medicines from BVBD were sent to PHO, from where they were sent to district, sub-district and health centers where it was repacked as single doses into small plastic sachets (a plastic sachet contained medicines required for one person) according to requirement for each IU. The repacked sachets for each IU were then sent to Village Health Volunteer (VHV), who implements various health programmes of the government at community level.

#### MDA: delivery channel

MDA was implemented every year in the month of April where 1 week in the month was designated as ‘Filaria week’ to actively implement the MDA programme. While some IUs completed MDA in 1 week, others required 2–3 weeks and the reports are completed in 4 to 8 weeks’ time. Depending upon population size, the number of VHVs employed per village mostly ranged from 5 to 10. Each volunteer was allocated a target of about 10–15 households. The health officers of the health centers supervised the drug distribution activity. The respective PHO closely monitored the drug distribution activity in each province. The policy of the programme is directly observed treatment where in every village the volunteer visited each household, provided the drug to each member and ensured treatment in his/her presence. Drugs were mostly distributed in the evening/night, between 18:00 and 21:00 h, and, as much as possible, treatment was given post-dinner to reduce the occurrence and severity of adverse events. In some provinces, drugs were administered also in some common places such as temples, community centres, leader’s house and mosque.

#### MDA: Eligible population

Children <2 years age and pregnant women and people with chronic disease conditions were excluded from treatment. All other groups were included for MDA and treatment. Drug dosage for each individual was determined based on age and weight and the medicines were given according to the age of the individuals.

#### Training cascade

Prior to launching the programme in 2002, the Division of LF provided training to the trainers, who included provincial level staff from the VBDC and PHO. Subsequently, they trained the staff at district level from the VBDU and DHO and health centers. The latter trained the VHV. Training was conducted at health centers for 1 day. The training was conducted every year, prior to the filaria week. Training focused on the objectives and goals of the LF elimination programme, transmission of LF and MDA programme that include details on medicines, dosage, exclusion and inclusion criteria, adverse events, social mobilization and microplanning.

#### Social mobilization

The LF elimination programme and MDA implementation was supported by an Information, Education and Communication campaign across all IUs in most of the target village emphasizing the importance of participating in MDA. In more endemic provinces, big events were held during the first or second day of Filaria Week with participation of Director-General of the DDC and other senior officers and officials from PHO.

#### Severe adverse events reporting and response

The VHV monitored the occurrence of adverse events (AEs) in the treated population drug distribution. Adverse events were reported by a health personnel through a national AE form that recorded any unusual symptoms for 2–3 days after ingestion of the drug. VHVs were also trained to inform those receiving the drug to report to the nearest health center if they had unusual symptoms. If those symptoms required further treatment, they were referred to the nearest district or provincial health facility for further management. However, throughout the MDA implementation period, the incidence of adverse events was negligible and there were no reports of any severe adverse event.

#### Recording and reporting

Structured household MDA forms were distributed at sub-district levels to VHV. The forms included all details of IU and name, age and gender columns for each household member and year-wise columns for drug distribution details. All the data were computerized in the Division of LF and hard copies retained. The denominator used for the calculation of epidemiological and national treatment coverage was the data from household forms which included the entire population of all age-groups which was updated every year.

This article describes the intensive MDA coverage in Thailand over the years from 2002 to 2011 and the subsequent impact of MDA through extensive monitoring and evaluation surveys – mainly through the Stop-MDA surveys and transmission assessment surveys. We also describe additional interventions through a LF chronic disease survey and outcomes of the LF survey among cats and LF surveillance among migrants that collectively enabled the validation of LF elimination in 2017. We also discuss briefly Thailand’s post LF elimination plans.

## Methods

### Monitoring and evaluation (M&E) surveys

**Fig. 3:**
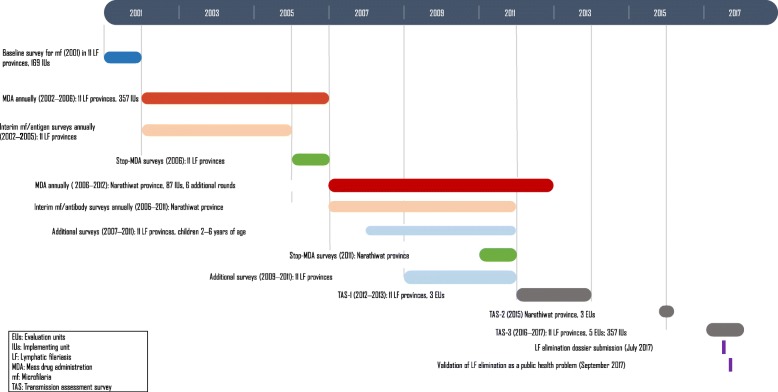
Timeline of key National Programme to Eliminate Lymphatic Filariasis activities in Thailand: 2001–2017

The programme undertook very extensive M&E surveys in three *W. bancrofti* endemic provinces with large number of IUs (Mae Hong Son, Tak and Kanchanaburi) and all the four *B. malayi* endemic provinces (Surat Thani, Krabi, Nakhon Si Thammarat and Narathiwat) to critically assess the impact of MDA. These seven provinces account for 346 of 357 IUs. The M&E consisted of (i) baseline mf surveys in 2001; (ii) interim (sentinel site or spot-check site) throughout the intervention period which consisted of mf surveys and antigenaemia surveys in *W. bancrofti* endemic provinces, and mf and antibody surveys in *B. malayi* endemic provinces (2002–2005 in ten provinces) and in Narathiwat (2002–2011); (iii) Stop-MDA surveys in 2006 in 11 provinces, and (iv) Transmission Assessment Surveys (TAS) over 2012–2017. Figure 3 summarizes the timelines of these surveys.

#### The baseline surveys (2001)

These were assessments of mf using thick blood smear examination performed during between 20:00 h and 24:00 h in *W. bancrofti* endemic IUs and *B. malayi* endemic IUs in Narathiwat, or daytime in *B. malayi* endemic provinces of Krabi, Surat Thani and Nakhon Si Thammarat. Base-line data were collected in a total of 169 IUs. The blood smears were stained with Giemsa and examined at VBDC or VBDU. In other IUs, data from surveys done prior to 2001 were analysed and areas with mf prevalence of more than 1% or where there was abundance of vector breeding sites, were included as LF endemic.

#### The interim surveys (2002–2011)

Interim surveys were done every year during 2002–2011, covering each year a proportion of IUs of the eleven endemic LF provinces. Mf prevalence (%), antigen prevalence (%) and antibody prevalence (%) were used as M&E indicators.

#### Stop-MDA surveys (2006)

It has been envisaged and showed that five rounds of effective MDA are likely to interrupt transmission of LF [9]. The surveys to stop MDA consisted of two components: (i) Assessment of antigenaemia or microfilaraemia in populations where surveys were conducted in the population age-groups of > 6 years of age. (ii) Assessment of antigenaemia in children of ≤6 years. Immunochromatographic test (ICT) test kits were used to detect *W. bancrofti* infection and mf blood smears for *B*. *malayi*,

#### Stop-MDA surveys in Narathiwat (2011)

In Narathiwat, where additional rounds of MDA over 2006–2012 were conducted, Stop-MDA surveys were done in 2011 and used a TAS methodology sampling children ≤6 years of age in 87 IUs. FilariaDIAG RAPID (an ELISA IgG4 test developed jointly by Mahidol and Chiang Mai hospital, Thailand) [10, 11]. In brugian endemic provinces, antibody assessment was introduced as soon as Brugia rapid test (BR) were available for the programme.

#### Additional surveys (2007–2011)

With encouraging results from Stop-MDA surveys in 2006 from the ten LF endemic provinces (excluding Narathiwat province), the programme focussed on child surveys in order to detect ongoing transmission, if any, and initiate remedial measures. These child surveys continued through 2007–2011 in ten of the 11 provinces in all IUs. Antigen surveys using immunochromatographic test (ICT) were conducted in *W. bancrofti* endemic provinces (*n* = 7, 489 IUs) and antibody surveys were conducted in *B. malayi* endemic provinces (*n* = 3, 15 IUs).

#### Transmission assessment surveys (TAS) (2012–2017)

As per WHO recommendations, two rounds of TAS was conducted after more than 4 years cessation of MDA [12]. TAS were school based, as the primary school enrolment rate was > 75%. Prior to every TAS, the BVBD approached and explained the objectives of the survey and sought the support of The Basic Education Commission, Ministry of Education (MOE). The MOE informed all the schools to participate in the surveys. The TAS teams contacted the principals of the schools and briefed them about the surveys, and provided an information sheet to all the parents highlighting the purpose and objectives of the survey. The written consent of the parents was obtained for each child. Refusal to consent was reported to be very rare. Within each evaluation unit (EU), the schools were selected and sample size determined using Survey Sample Builder (https://www.ntdsupport.org/resources/transmission-assessment-survey-sample-builder).

The first surveillance, TAS-1 (2012–2013) was conducted in all IUs of 11 provinces which were regrouped into three EUs (EU-1, EU-2 and EU-3). In TAS-2 (2015), the survey was done only in Narathiwat province where the 87 IUs grouped in EU-3 were further reorganized to 3 separate EUs – EU-3.1 (18 IUs), EU-3.2 (32 IUs) and EU-3.3 (37 IUs) to ensure robust evaluation of incidence of infection among children. In TAS-3 (2016–2017), all five EUs were surveyed. The impact indicator used in TAS was antigenaemia incidence and prevalence in *W. bancrofti* endemic areas and antibody incidence and prevalence in *B. malayi* endemic areas among pre-school, 1st and 2nd grade school students, most of who are in 6–7 year age-group. The antigenaemia prevalence was measured using ICT card tests during the TAS-1 and 2 and Filaria Test Strip (FTS) during TAS-3. The antibody prevalence was measured using the BR. Children showing positive result for antibody test done during the day time in *B. malayi* endemic areas, were blood tested also for mf during the night. All children with antigenaemia or antibody positive were treated with full course of DEC. DEC was given six monthly thereafter for two consecutive years. In *B. malayi* endemic provinces, if any antibody positive person was found with mf, a contact survey was undertaken. All members of about 15 households close to the mf positive household were blood tested for mf and if found to be positive, a full course of DEC was administered.

#### LF chronic disease survey

At the commencement of preparations for the MDA in 2001, the program initiated in parallel to epidemiological surveys, a LF chronic disease survey as part of its strategy for Morbidity Management and Disability Prevention (MMDP). The health workers of the health centers of the sub-district in LF endemic areas assessed the presence of patients affected with lymphoedema/elephantiasis or hydrocele during regular house visits and when blood surveys were conducted. A list of chronic disease patients was prepared for each health center in each province and updated every year. The health workers visited all the households with patients. They were trained to provide care as well as education to the patients and family members on leg hygiene and the patients were followed up for 2 months to support them in the practice of leg hygiene. The patients were given a MMDP kit that contained soap, cotton, bandage, anti-fungal ointment (Clotrimazole), gauze cloth, towel and anti-septic solution (Ipodine). Some patients were also provided with elastic stockings. Each patient was also given a booklet with pictures of MMDP steps. These kits were provided once to the patient with instructions that if an episode of acute dermatolymphangioadenitis (ADL) occurs, the patient could approach the nearest health facility to obtain supportive medication such as paracetamol for fever, ipodine for wound dressing, pressure bandage for lymph circulation etc. The patients were advised to consult doctors for the treatment of ADL attacks and any other related complications. Hydrocele surgeries are provided in provincial hospitals and although such cases are rare, necessary infrastructure, medicines and follow-up services to undertake hydrocele surgery are available in all the provincial hospitals.

## Results

### Delineation of endemicity

**Fig. 4:**
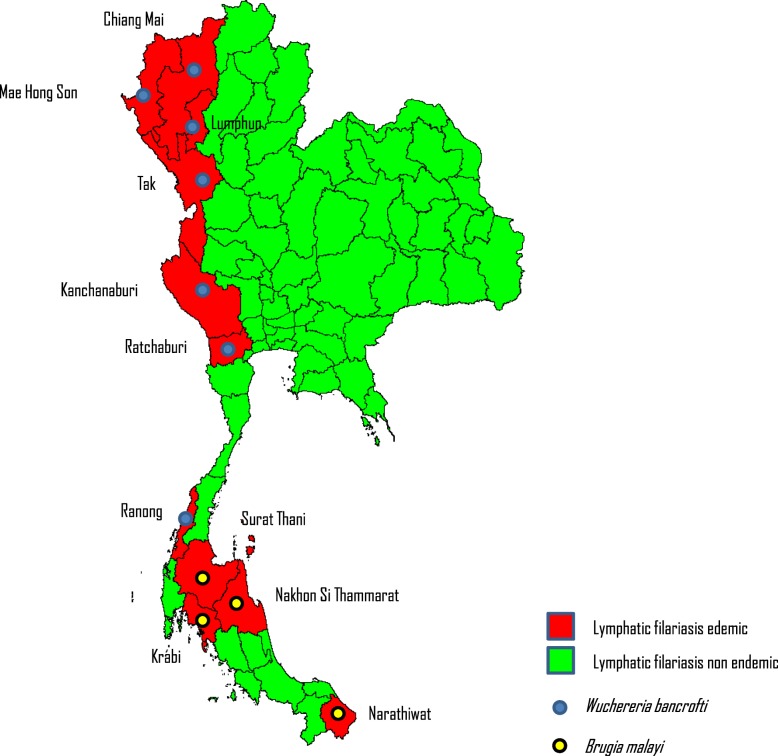
Delineation of Lf endemicity at the commencement of the National Programme to Eliminate Lymphatic Filariasis in Thailand, 2001

Within the 11 provinces, 357 sub-villages, were declared endemic for LF and eligible for the MDA programme (see Table 1). The total population of the 357 sub-villages in 2002 was 124 496. Although all 11 provinces are endemic, four of these provinces – Mae Hong Son, Tak, Kanchanaburi and Narathiwat, accounted for 336 of 357 (94%) of endemic sub-villages. In the same 11 provinces, a total of 283 villages were excluded from MDA, as the mf and/or antigen prevalence was below threshold levels of 1.0 and 2.0% respectively. The sub-village administrative unit was designated as an IU. The average population of an IU was 349. Figure 4 shows the 11 LF endemic provinces mapped by the causative vector species. All IUs in the following were endemic for *W. bancrofti* (seven provinces), transmitted by *Aedes niveus*; and *B. malayi* (4 provinces), transmitted by *Mansonia* species. The *W. bancrofti* endemic provinces are located in north and central Thailand, *B. malayi* endemic provinces are in south Thailand.

**Table 1:** Number of sub-villages identified as endemic in eleven lymphatic filariasis (LF) endemic provinces and population in 2002

Province	Number of sub-villages identified as LF endemic	Total population in identified sub-villages (2002)
Mae Hong Son	76	11 257
Chiang Mai	2	370
Lamphun	3	2436
Tak	124	31 200
Ratchaburi	4	787
Kanchanaburi	49	19 179
Ranong	2	4405
Surat Thani	6	4073
Nakhon Si Thammarat	2	710
Krabi	2	672
Narathiwat	87	49 407
Total	357	124 496

### MDA as major intervention for LF elimination

The mean MDA coverage for the entire country from 2002 to 2011 was 90.5% (68 to 95.6%), Table 2. Due to the unrest in the south of Thailand, only 15 out of 87 IUs in Narathiwat could be covered with MDA. Stop-MDA surveys were done in the 15 IUs and although *B*. *malayi* mf prevalence was 0.2%, a decision was made to continue with more rounds of MDA. In one endemic province, Narathiwat (87 IUs), MDA had to be extended until 2012 for a total of 11 rounds due to persistent infection.

**Table 2:** Summary of national mass drug administration coverage during 2002–2011

Year	Number of IUs covered	Geographical coverage (%)	Total population of IUs	Reported number of people treated	Programme (drug) coverage (%)
2002	340	100%	124 496	114 955	92.3%
2003	344	100%	147 177	131 346	89.2%
2004	345	100%	152 111	131 393	86.4%
2005	341	98.0% (341/348)	166 636	144 252	86.6%
2006	308	88.3% (308/349)	166 647	113 380	68.0%
2007	75	91.5% (75/82)	77 935	61 776	79.3%
2008	87	100%	79 267	75 671	95.5%
2009	87	100%	81 601	77 842	95.4%
2010	87	100%	81 098	76 707	94.6%
2011	87	100%	83 105	79 210	95.3%

*IUs* Intervention units

#### The baseline surveys (2001)

Of the 169 surveyed IUs in 2001, 129 IUs showed < 1.0% mf prevalence, the threshold level at which LF transmission is unlikely to sustain.

#### The interim surveys (2002–2011)

The MDA programme was launched in the year 2002 and was implemented annually in consecutive years from 2002 to 2006 in endemic IUs of 11 provinces involving a total of 357 IUs with a total population of 124 496 (year 2002). Interim surveys were done every year during 2002–2011 and results (Tables 3 and 4) suggest that, with the progress of MDA, by 2005, only one IU, in Tak province, showed >2.5% antigen prevalence. Almost all IUs in *B. malayi* endemic provinces, except Narathiwat province, showed <1.0% mf prevalence and by 2005, mf carriers had become rare. In Narathiwat, endemic for *B. malayi*, as most IUs showed prevalence of microfilaraemia consistently >1.0% until 2005, the programme decided to continue MDA for few more years. A total of 11 rounds of MDA were implemented in this province (2002–2012). The continuous interim surveys in Narathiwat province subsequently showed that in 2006, 2008 and 2009, the highest mf prevalence recorded was only 0.8% in the community (population of all age-groups).

**Table 3:** Details of interim surveys done in provinces endemic for *Wuchereria bancrofti*

Province	Year	Age group	Antigen survey^a^								Microfilaria (mf) survey							
			Number of IUs surveyed	Total population in IUs surveyed	Total number of people tested in IUs surveyed	Number of IUs with positive result	Total number of population in IUs where found positive	Total number of people tested in IUs where found positive	Positive case	Highest prevalence observed in IUs surveyed (range)	Number of IUs surveyed	Total population in IUs surveyed	Total number of people tested in IUs surveyed	Number of IUs with positive result	Total number of population in IUs where found positive	Total number of people tested in IUs where found positive	Positive case	Highest prevalence observed in IUs surveyed (range)
Mae Hong Son	2002	All	0	0	–	0	0	0	0	0	15	2724	2122	2	509	509	9	2.4% (1.0–2.4%)
	2003	All	14	2485	1733	2	318	245	5	3.2% (0.8–3.2%)	1			1	84	52	0	1.9%
	2004	All	12	1909	1538	0	0	0	0	0	0	–	0	0	0	0	0	0
	2005	All	3	651	403	0	0	0	0	0	8	1182	753	0	0	0	0	0
Chiang Mai	2004	All	0	0	0	0	0	0	0	0	1	977	159	0	0	0	0	0
	2005	All	1	646	31	0	0	0	0	0	1	1240	326	0	0	0	0	0
Lamphun	2003	All	0	0	0	0	0	0	0	0	2	2985	655	0	0	0	0	0
	2004	All	0	0	0	0	0	0	0	0	2	2072	738	0	0	0	0	0
	2005	All	1^b^	982	75	0	0	0	0	0	1	982	623	0	0	0	0	0
Tak	2002	All	20	5694	4242	2	903	408	3	2.6% (0.3–2.6%)	1	458	46	0	0	0	0	0
	2003	All	26	6034	4795	2	194	145	6	10.4% (1.0–10.4%)	6	2535	1840	0	0	0	0	0
	2004	All	13	2496	2786	6	1671	1283	13	6.7% (0.2–6.7%)	3	632	382	0	0	0	0	0
	2005	All	8^b^	2386	240	1	331	284	1	0.4%	11^b^	3145	2358	0	0	0	0	0
Ratchaburi	No survey data																	
Kanchanaburi	2002	All	38	14 410	9311	6	3497	2292	28	2.2% (0.5–2.2%)	0	0	0	0	0	0	0	0
	2003	All	8	2991	2523	1	240	164	11	6.7%	4	1342	1319	0	0	0	0	0
	2004	All	8	3575	2696	0	0	0	0	0	6	1152	982	0	0	0	0	0
	2005	All	12	4524	543	2	327	252	6	2.5% (2.2–2.5%)	10	4197	3546	0	0	0	0	0
Ranong	No survey data																	

*IUs*: Intervention units

^a^Antigen assessment, which is more sensitive than mf assessment, could not be adopted in every survey due to immunochromatographic test (ICT) card supply constraints

^b^These IUs used both ICT card & mf detection

**Table 4:** Details of interim surveys done in provinces endemic for *Brugia malayi*

Province	Year	Age-group surveyed	Number of IUs surveyed	Total population in IUs surveyed	Total number of people tested in IUs surveyed	Number of IUs with positive result	Total number of population in IUs where found positive	Total number of people tested in IUs where found positive	Number of positive cases	Highest prevalence observed in IUs surveyed (range)
Microfilaria (mf) surveys										
Surat Thani	2002	All	4	2580	1447	4	2580	1447	5	10.0% (0.2–10.0%)
	2003	All	1	587	454	0	0	0	0	0
	2004	All	1	591	475	0	0	0	0	0
	2005	All	2	1585	978	1	632	549	2	0.4% (0–0.4%)
Nakhon Si Thammarat	2002	All	1	613	609	0	0	0	0	0
	2003	All	2	777	757	0	0	0	0	0
	2004	All	2	879	741	1	747	624	2	0.3% (0–0.3%)
	2005	All	2	873	672	0	0	0	0	0
Krabi	2002	All	1	425	403	0	0	0	0	0
	2003	All	2	653	382	0	0	0	0	0
	2004	All	2	654	420	0	0	0	0	0
	2005	All	1	431	362	0	0	0	0	0
Narathiwat	2002	All	28	15 501	11 791	**23**	13 125	11 119	84	3.5% (0.2–3.5%)
	2003	All	25	18 873	12 852	15	10 372	7211	32	1.6% (0.2–1.6%)
	2004	All	20	14 072	9239	9	7281	4683	18	1.1% (0.1–1.1%)
	2005	All	24	18 373	10 097	**16**	11 872	6490	33	1.6% (0.1–1.6%)
	2007	All	34	28 557	17 512	15	15 670	8589	19	2.1% (0.1–2.1%)
	2008	All	35	29 720	18 931	5	3982	2295	5	0.5% (0.2–0.5%)
	2009	All	23	19 975	14 000	6	6958	4642	13	0.5% (0.1–0.5%)

*IUs* Intervention units

#### Stop-MDA surveys (2006)

Antigenaemia and mf prevalence in *W. bancrofti* endemic provinces among populations > 6 years of age was 0% in all evaluated IUs with only 1 IU showing mf prevalence of 2.7% (Table 5). In three of the *B. malayi* endemic provinces (excluding Narathiwat), mf prevalence among populations > 6 years of age in 10 IUs surveyed was 0%. In Narathiwat, out of 15 IUs surveyed, nine mf positive cases were detected in seven IUs with the highest mf prevalence of 0.8% (range: 0.1–0.8%). Assessment of antigenaemia and mf in children of ≤6 years showed that no child was found positive for antigen in any *W. bancrofti* IU in any province, and no child was found positive for mf in *B. malayi* in the three endemic provinces. The Stop-MDA surveys suggested that by 2006, transmission of LF and incidence of new infections had become very rare in the ten LF endemic provinces.

**Table 5:** Stop mass drug administration (MDA) surveys

Antigen detection	Filarial parasite	Number of provinces	Total number of IUs	Number of IUs surveyed	Test used	Result
Stop MDA survey, 2006						
> 6 years of age	*Wuchereria bancrofti*	5	206	20	ICT	206 IUs: 0%
		2	54	26	mf	1 IU: > 2.7%
	*Brugia malayi*	4	97	25	mf	7 IUs: 0.3% ^a^
≤ 6 years of age	*W. bancrofti*	7	260	232	ICT	0%
	*B. malayi*	4	97	24	mf	0%
Stop MDA survey – Narathiwat province, 2011						
≤ 6 years of age	*B. malayi*	1	87	87	ELISA^b^	71 IUs: 0% 3 IUs: < 2%^c^ 13 IUs: > 2%^c^

*ICT* Immunochromatographic test, *IUs* Intervention units, *Mf* Microfilaria

^a^ *Out of 25 IUs, 15 IUs surveyed were in Narathiwat province. Nine mf positive cases were detected in seven IUs in Narathiwat province with, highest mf prevalence of 0.8% (0.1–0.8%)*

^b^*FilariaDIAG RAPID (an ELISA IgG4test developed jointly by Mahidol and Chiang Mai hospital, Thailand)*. *In Brugia endemic provinces, antibody assessment was introduced as soon as Brugia rapid test were available for the programme*

^c^ *Antibodies detected in 16 IUs among 26 children [Antibody rate: 33.3% (11.0–33.3%) who were subsequently tested for mf of whom seven were positive [mf positivity rate: 4.2% (0.4–4.2%). ELISA test was used during the day time for children. Positive ELISA cases were subsequently tested for mf during the night*

#### Stop-MDA surveys in Narathiwat (2011)

Following the last round of MDA in Narathiwat, a Stop-MDA survey was done in 2011. Of the 87 IUs surveyed, antibodies were detected among 26 children in 16 IUs who were subsequently tested for mf of whom seven were positive, and the mf positivity rate ranged from 0.4 to 4.2%.

#### Additional surveys (2007–2011)

Child surveys continued through 2007–2011 in ten of the 11 provinces in all IUs. None of the sampled children was found positive either for antigen or antibody among a number of IUs surveyed during different years, indicating near total interruption of transmission.

#### Transmission assessment surveys (TAS) (2012–2017)

The coverage and results of both TAS-1 to TAS-3 are shown in Table 6. In TAS-1 (2012–2013), the number of children positive for antigen or antibody was much lower than critical cut off value in both EU-1 and EU-2, and no child was found to be mf positive in EU-2 which clearly indicated that transmission was completely interrupted in both the EUs. In Narathiwat province (EU-3) which had historically higher prevalence rates and required more MDAs, TAS-1 sampled 1018 children against the target of 1356 in all the 87 IUs. The under sampling was due to some unrest incidents in the province involving schools. Antibody prevalence was 0.7% with seven children positive, against the critical cut off value of 16. Of the seven antibody positive children, two were mf positive. The results suggest that LF transmission was well below threshold levels in Narathiwat by 2013. In TAS-2 (2015), EU-3 was reorganized into three EUs and the number of children found positive for antibody was either equal or below the critical cut off value signifying transmission below threshold level. Two of the 11 antibody positive children showed mf in the blood. The same EU grouping was continued for TAS-3 (2016–2017) in all 11 provinces (Table 6). None of the tested children were found positive except for EU 3.3, where four of the 530 children were tested positive for antibody, against the critical cut off value of six. This indicated that transmission was totally interrupted in all five EUs. In all the three TAS, contact screening of mf positive children were done among household members of about 15 households around each of the mf positive child, and none of the household members were found positive.

**Table 6:** Transmission Assessment Surveys (TAS) 2012–2017: Coverage and results

Year	Number of provinces	Number of EUs	Number of IUs	Number of children tested	Species^a^/Test	Number children positive for antigen or antibody ^b^/ critical cut off value	Number of mf positive children ^c^	Contact survey result
2012–	10	EU-1	260	1786	WB/Ag	0 /11	0	–
2013 (TAS-1)		EU-2	10	129	BM/Ab	1 / 2	0	–
		EU-3	87	1018	BM/Ab	7/16	2	Negative
2015 (TAS-2)	1	EU-3.1	18	284	BM/Ab	2 / 3	0	–
		EU-3.2	32	506	BM/Ab	3 / 6	0	–
		EU-3.3	37	506	BM/Ab	6 / 6	2	Negative
2016–2017 (TAS-3)	11	EU-1	260	2233	WB/Ag ^d^	0 /11	0	–
		EU-2	10	144	BM/Ab	0 / 2	0	–
		EU-3.1	18	284	BM/Ab	0 / 3	0	–
		EU-3.2	32	506	BM/Ab	0 / 6	0	–
		EU-3.3	37	530	BM/Ab	4 / 6	3	Negative

*EUs* Evaluation units; IUs: Intervention units

^a^ *WB: Wuchereria bancrofti; BM: Brugia malayi*

^b^ *Antigen (Ag) with immunochromatographic test (ICT); Antibody (Ab) with Brugia Rapid test*

^c^ *Among Ag or Ab positive children*

^d^ *Filaria Test Strip (FTS) was used instead of ICT*

#### LF chronic disease survey

The number of lymphoedema/elephantiasis patients detected during different years in Thailand is shown in Table 7. An update registry maintained at BVBD as of April 2017 showed a total of 99 patients followed-up under 34 health centers, of which, a total of 69 patients (70%) were under the care of 14 health centres in just one province of Nakhon Si Thammarat.

**Table 7:** Number of lymphoedema/elephantiasis patients detected during different fiscal years (FY) in Thailand

Province	FY 2001	FY 2005	FY 2006	FY 2007	FY 2014	FY 2015	FY 2016
Chumphon	10	10	10	7	6	6	3
Surat Thani	31	25	25	18	13	13	9
Nakhon Si Thamrat	181	181	181	162	125	86	69
Krabi	1	0	0	0	1	1	1
Phangnga	2	3	3	1	0	0	0
Phatthalung	13	13	13	9	6	6	1
Pattani	17	26	26	19	17	17	9
Narathiwat	29	33	33	29	17	17	8
Total	284	291	291	245	185	146	100

### Special issues

#### LF in cats

As early as the late 1980s, cat surveys documented *B. malayi* and *B. pahangi* infection among domestic cats in all four *B*. *malayi* endemic provinces of Surat Thani, Nakhon Si Thamarat, Krabi and Narathiwat. LF infection was not found in other animals such as dogs and monkeys [5, 13]. To interrupt zoonotic transmission, beginning in 2003, active surveillance of cats in areas with > 1.0% mf rate among cats was done along with mass treatment of cats with ivermection given subcutaneously. In addition, in every area, all the cats found with LF infection were treated with ivermectin annually. As a result of this intensive treatment, the mf prevalence among cats declined from 8.1% in 1995 to as low as 0.8% in 2015. Post elimination surveillance in Narathiwat and other *B. malayi* endemic provinces will continue to perform cat surveys and treatment along with ongoing surveillance in human population to prevent possible zoonotic transmission of LF in Narathiwat.

#### LF surveillance among migrants

Attapeu province in Lao Peoples Democratic Republic is endemic for LF but it shares no immediate geographic border or significant population movement with Thailand [14]. The Preah Vihear province of Cambodia, which was endemic for LF, borders Thailand but has since achieved validation of elimination of LF in the year 2016. Although four border states of Kedah, Kelantan, Perak and Perlis in Malaysia border the provinces of Narathiwat, Satun, Songkhla and Yala, these states had achived LF elimination with only Perak receiving 5–7 rounds of MDA and passed TAS-1 by 2016 [15].

However, Thailand shares a very long border with Myanmar, several provinces of which are endemic for LF caused by *W. bancrofti* and transmitted by *Culex quinquefasciatus*. Although there has been some debate on human-vector combinations on the risk of *W. bancrofti* transmission across the Thai-Myanmar borders [16, 17], current data/information thus far is not sufficient to understand the vulnerabilities on how contagious is the parasite in such complex epidemiological settings as well as the receptivity of the vector in different ecological settings along the borders [18]. Numerous studies among Myanmar migrants [19–22] in Thailand, prior to intensive MDA campaigns in Myanmar, documented higher prevalence of antigens and antifilarial antibodies among Myanmar immigrants [23]. Since 2001, the Thai MoPH set up the migrant health insurance scheme for all migrants (documented and undocumented) who are not covered by social health insurance, allowing mandatory health screening (during the first entry and subsequent yearly renewal of the residence permit) [24] which includes testing for bancroftian mf (mf challenge test with DEC) which is done at all district hospitals and for which a full course of treatment (single dose of DEC + ALB) is offered if found to be positive.

In addition, in a number of provinces (average: 19, range: 13–25) where there were significant number of migrant workers registered, sentinel site surveillance for mf was done annually between 1996 and 2001 with a total of 204 108 persons tested with a blood film for mf yielding an average positivity rate of 0.7% (range: 0.2–2.2%) over the same period [25]. With the commencement of the NPELF, the annual surveillance of migrants was focused on seven provinces over 2002–2017 with 23 477 persons tested for LF antigen using ICT test cards, showing a positivity rate of 0.7% (range: 0.1–2.7%) over the same period. Where antigen positivity was detected among migrants in these areas, the Thai populations residing in close proximity were also concurrently tested over the same period (average 2616) with zero positivity rates. In addition, the local health facilities are encouraged to treat the immigrant population regardless of legal status. Both measures, the MDAs conducted in Myanmar [26] and the screening and treatment of migrants in Thailand [27–29], were probable contributors to the decline in the number of LF cases detected in Thailand among Myanmar migrants.

## Discussion

Elimination of LF as a public health problem is defined as reduction in measurable prevalence of infection in endemic areas below a target threshold at which further transmission is considered unlikely even in the absence of MDA [30]. These target thresholds are measured during TAS. However, a programme must first achieve <1% microfilaraemia or < 2% antigenaemia among populations aged older than 5 years in sentinel and spot-check sites considered high-risk. Then, all endemic areas should pass TAS (the number of positive children is less than the critical cut-off value indicating infection is below elimination thresholds) and stop MDA. Infection must be maintained below these levels for at least 4 years after MDA has stopped.

### Role of government

Since the establishment of the Division of Lymphatic Filariasis in 1961 under the Department of Health of the Royal Thai Government ensured that resources were allocated for national surveys to identify the endemic areas, followed by integrated vector control efforts, continuous entomological and parasitological surveillance efforts, and repeated annual rounds of high coverage MDAs among at-risk groups. With the establishment of the universal health coverage (UHC) scheme in 2001 and subsequently migrant health insurance schemes, the provision of free morbidity management and disability prevention services were extended to the sub-district Tambon Health Promotion Hospital and for both registered and unregistered migrants. The Regional LF offices (five regions) established in the in the 1970s were instrumental in overseeing case finding, treatment and entomological surveys under LF control program. The Thai Royal Filaria Project established the Phikulthong Royal Development Study Center in Narathiwat province provided all necessary support with infrastructure and required personnel for LF control, and subsequently elimination efforts in Narathiwat Province.

### Partnerships

Thailand has partnered with WHO and national universities such as Mahidol University especially in the early surveys undertaken in the 1950s to the 1960s. ALB was provided by GlaxoSmithKline through the WHO donation program. DEC (50 and 300 mg) was procured by the program from a local pharmaceutical manufacturer. Eisai Co., Ltd. provided on request in 2015, for 100 mg tablet of DEC to be used in children. WHO SEARO assisted with the procurement of ICT, FTS and BR for TAS as well as training programmes for LF patients. Excellent support was provided by the Ministry of Education through its Basic Education Commission, for TAS in school children.

### Validation

Based on the MDA coverage data, TAS results and the established MMDP services, a country dossier was prepared under the guidance of the Regional Programme Review Group (RPRG). The dossier documented sufficient evidence that Thailand has met the established criteria to validate elimination of LF as a public health problem. An independent Regional Dossier Review Group convened by WHO reviewed the dossier in mid-2017 and endorsed that the elimination criteria was met. Based on this evidence, WHO validated and formally acknowledged that the Kingdom of Thailand has eliminated LF as a public health problem in September 2017.

### Post validation surveillance

The RPRG in its review recommended that Thailand continue post-MDA activities monitoring for infection in migrant population and zoonotic hosts of *B. malayi*. It also recommended continuing soil-transmitted helminthiases (STH) control activities in the areas at risk. A health facility survey is planned to be conducted every 2 years from 2017 in all areas with LF patients. The quality of services provided to the patients will be assessed through the following methods: (1) availability of medicines; (2) assessment of the number of patients with ADL and lymphoedema visiting the health facility; and (3) interview with health personnel on the treatment provided to patients and (4) interview with patients to assess their opinion on services provided and improvement in quality of life. The data collected by visiting the health centers will be analyzed and corrective steps, if necessary, will be taken to improve the services. Targeted surveillance will continue every 2 years in all previous ten LF endemic provinces with a coverage of 10% of total IUs in each province. All populations in these IUs will be blood surveyed. In Narathiwat province this will be done annually in 10% of total IU. Vector surveys will be conducted in 1% of total IUs in each province. In migrants, both routine health screening for migrant workers and blood spot checks annually and mosquito surveys will continue in communities in the ten LF endemic provinces depending where there are high influx or movement of migrant workers. Additionally, in Narathiwat province, surveillance among cats will be conducted periodically.

## Conclusions

From the baseline survey in 2001, the LF elimination programme in Thailand represents a typical post-control low endemic situation (i.e. a few decades of mf surveys and test and treatment strategy prior to commencing a LF elimination programme). The approach of the programme in affected provinces to adopt a sub-village as the IU also ensured a smaller population size and thus achieve better social mobilization efforts and compliance in taking annual doses of the medication even though they showed no symptom of disease. The interim surveys throughout the MDA period showed that mf and antigen prevalence was restricted to only a few IUs falling below threshold level in a significant number of IUs (except in Narathiwat province) by 2005. In highly endemic province of Narathiwat, IUs were reorganized to three separate EUs during TAS to ensure robust evaluation of incidence of infection among children. Sustained commitment by the government and dedicated health staff on the ground throughout the elimination phase, not only ensured the NPELF objectives were met finally in 2017 but also ensuring that high quality of care be continued for chronic LF patients. Along with post validation surveillance efforts every 2 years, the program will continue its LF surveillance efforts especially among migrant populations along provinces bordering Myanmar. The Phikulthong Royal Development Study Center in Narathiwat will continue monitoring for zoonotic LF transmission while focusing on STH and Leprosy control as well.

## Additional file

Additional file 1: Multilingual abstracts in the five official working languages of the United Nations. (PDF 691 kb)

## Abbreviations

ALB: Albendazole
BVBD: Bureau of Vector Borne Diseases
DDC: Department of Disease Control
EU: Evaluation unit
GDP: Gross domestic product
GPELF: Global Programme to Eliminate Lymphatic Filariasis
GSK: Glaxo-SmithKline
ICT: Immunochromatographic Test
IU: Intervention unit
LF: Lymphatic filariasis
M&E: Monitoring and evaluation
MDA: Mass drug administration
Mf: Microfilaria
MMDP: Morbidity Management and Disability Prevention
MoPH: Ministry of Public Health
NPELF: National Programme to Eliminate LF
NTD: Neglected Tropical Diseases
PHO: Provincial Health Office
SDHC: Sub-district Health Centre
SEARO: South-east Asia Regional Office
STH: Soil-transmitted helminthiases
TAS: Transmission Assessment Survey
UHC: Universal health coverage
VBDC: Vector Borne Disease Centre
VBDU: Vector Borne Disease Unit
VHV: Village Health Volunteer
WHO: World Health Organization
