# Correction to: How Thailand eliminated lymphatic filariasis as a public health problem

**DOI:** 10.1186/s40249-019-0582-0

**Published:** 2019-08-16

**Authors:** Sunsanee Rojanapanus, Tanaporn Toothong, Patcharida Boondej, Suwich Thammapalo, Naraporn Khuanyoung, Weena Santabutr, Preecha Prempree, Deyer Gopinath, Kapa D. Ramaiah

**Affiliations:** 1 Bureau of Vector Borne Diseases, Department of Disease Control, Ministry of Public Health, Nonthaburi, Thailand; 2 Office of Disease Prevention and Control, Ministry of Public Health, Songkhla, Thailand; 3 World Health Organization, Country Office for Thailand, Nonthaburi, Thailand; 4 Consultanton lymphatic filariasis, Tagore Nagar, Pondicherry, India

**Correction to: Infect Dis Poverty (2019) 8:38**

**https://doi.org/10.1186/s40249-019-0549-1**

After publication of this article [1], it was brought to our attention that Fig. 2 did not display correctly. The correct Fig. 2 is as below:

**Fig. 2:**
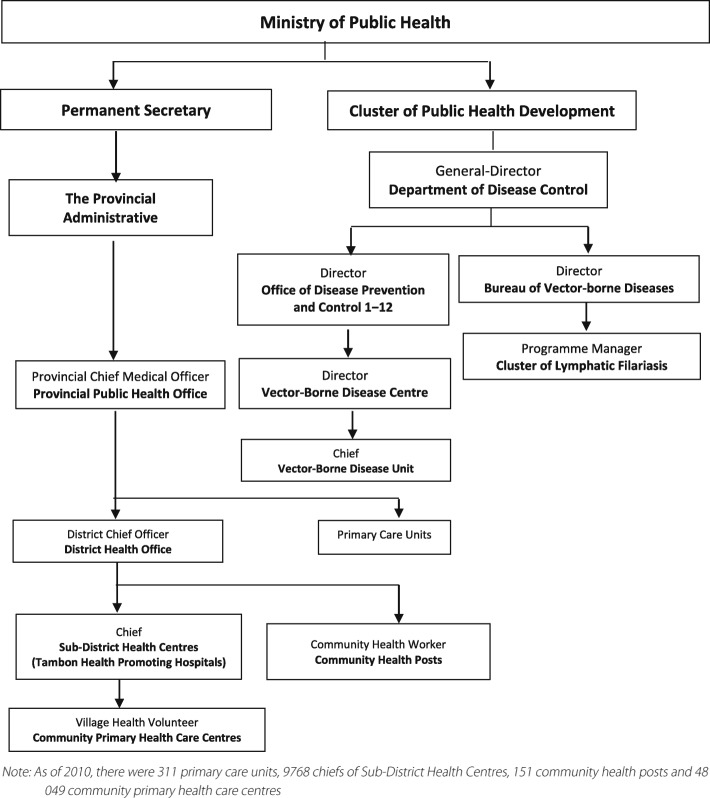
Structure of National Programme for Elimination of Lymphatic Filariasis in Thailand
