# Supplementary material for: How Thailand eliminated lymphatic filariasis as a public health problem
Source: Infect Dis Poverty. 2019 May 27;8:38. doi: 10.1186/s40249-019-0549-1 (PMC6535972; doi:10.1186/s40249-019-0549-1)
Supplement: Supplementary file 1 — Multilingual abstracts in the five official working languages of the United Nations. (PDF 691 kb) [file 40249_2019_549_MOESM1_ESM.pdf]

Translation of the abstract into the five official working languages of the United Nations

عامه صحه يةكمشكله الـ لمـ فاوية الـ ف يلاريات داء ت ايـ لـ ند ألـ غت ك يف

، ساند تابـ وو ويـ نا ، خواند يونـ غ نـ ارابـ ورن ، ثـ امابـ الـ و سويـ تش ، بـ و نـ ديـ ج بـ اتـ شاريـ دا ، مـ سواك تـ انـ ابـ ورن ، Sunsanee Rojanapanus
رادايـ اه .د Kapa ، غوبـ يـ ناث ديـ ايـ ر ، بـ ريـ مـ بري بـ ريـ تـ شا

مجرده

منطقه في الـ عالـ مـ ية الـ صحة منظمه في عشره الاحدي الأعـ ضاء الـ دول من تـ سع في مـ توطن الـ لمـ فاوي الـ ف يلاريـ ات داء :الـ خـ لـ فـ ية في الـ لمـ فاوية الـ ف يلاريـ ات داء عـ لي لـ لـ قضاء نـ يالـ وطـ الـ برنامج مع الـ مكـ ثـ فة الـ تدخلات الـ مقالة هذه تـ و ضح .ا سـ يا شرق جـ نوب 2017 في عـ لـ يه الـ قضاء من الـ تحقق حـ تى 2001 في إطلاقه منذ تـ ايـ لانـ د.

الـ مـ توحـ شة الـ خـ يطـ ية الـ خطوط فـ يها كانـ ت الـ تي الـ مـ ناطق لـ تحديد 2001الـ في أ سا سـ يه وبـ ائـ ية ا سـ تـ قصائـ ية درا سة بـ دات :الـ طرق (DEC) كاربـ امازيـ ن ايـ ثـ يل ثـ نائـ ي سـ يـ ترات مع (MDA) لـ لادويـ ه ملةالـ شا الادارهـ تـ نـ فـ يذ تـ م .حدودها ورسمت والـ قاعديـ ة (LF). الـ لمـ فـ ية الـ ف يلاريـ ات بـ داء الـ موبـ وءة الـ مـ قاطعات من 11 في (IUs) تـ نـ فـ يذ وحده 357 مجموعه ما في (ALB) والالـ بـ يـ ندازول 11 الـ مـ توطـ نة لـ مـ قاطعاتا في 2006 في MDA لـ وقـ ف الا سـ تـ قصائـ ية الـ درا سات وأجريـ ت .فـ رعـ يه قـ ريـ ة (IU) الـ مـ نـ فذة الـ وحدة وكانـ ت مـ سـ تضد عن لـ لـ كـ شف (ICT) الا سـ تـ شراب اخـ تـ بار بـ ا سـ تخدام سـ نوات 6 ≤ من والأطـ فال الـ عمر من سـ نوات 6 من أكـ ثر الـ سكان بـ ين LF بـ ا سـ تخدام 2011 في MDA لـ وقـ ف مـ سوحات اجراء تـ م ، نـ اراتـ يوات مـ قاطعه في .الـ ملايـ و بـ روجـ يا (mf) الـ صغـ يرة والـ مـ يكروبـ ات 6 الـ عمريـ ة الـ فـ ئة في الـ مدارس طلاب بـ ين 2016-2017 و2015 ,2012-2013 في (TAS) الإر سال مـ تـ قـ يي مـ سوح أجريـ ت .ELISA -2002 عام خلال مـ قاطعات سـ بع في لـ لمهاجريـ ن الـ وطـ ني الـ صحي الـ فحص خلال من الـ مهاجريـ ن الـ سكان مراقـ بهوكـ ثـ فت .سـ نه u20127 الـ مـ ناطق من مـ قاطعات أربـ ع وفـ ي .والاتـ صالات الـ معـ لومات تـ كـ نولـ وجـ يا اراخـ تب بـ طاقـ ات بـ ا سـ تخدام بـ الـ دم الـ تـ سمم لـ مكافـ حه 2017 درا سة إذ شات وقـ د .1994 في الـ محـ لـ ية الـ قطط في LF خزان عن لـ لـ كـ شف الـ سـ نويـ ة الا سـ تـ قصائـ ية الـ درا سات بـ دات ، بـ ها الـ موبـ وءة داء/الـ لمـ فـ ية الـ وذمه من انـ ونـ يـ ع الـ ذيـ ن لـ لأ شخاص الـ تراكمي لـ لعدد سجلا LF لـ بـ الـ نـ سـ بة الـ مزمـ نة الامراض لـ عبء 2001 ا سـ تـ قصائـ ية الـ فـ نطـ يات.

جولات إلـ ى حاجه هناك وكانـ ت .IUs جمـ يع في 2002-2006 مدي عـ لي سـ نويـ ا MDA من جولات خمس مجموعه ما تـ نـ فـ يذ تـ م :الـ نـ تائـ ج تالمخدرا تـ غطـ يه وكانـ ت .الـ مـ سـ تمرة الـ عدوى بـ سـ بب 2007-2011 من نـ اراتـ يوات مـ قاطعه من 87 IUs في MDA من ا ضافـ يه سـ نويـ ة 11 الـ مـ قاطعات في 2006 في MDA وقـ ف الـ مـ سوحات .٪ 95.4 إلـ ى ٪ 68.0 نـ طاق في 2002-2012 عـ لي MDA مع الـ سـ نويـ ة الـ وطـ نـ ية 0.1 :الـ مدى) ٪ 0.8 انـ تـ شار معدل اعـ لي مع نـ اراتـ يوات مـ قاطعه في IUs سـ بعه في mf الايـ جابـ يه الـ حالات تـ سعه وجدت الـ مـ توطـ نة LF الـ مـ ضادة الأجـ سام بـ ين و سطـ الـ ملايـ و .bلـ الإر سال عـ تـ به معدلات أدنـ اه كـ شـ فت TAS-3 و 2-تـ اس ، 1-تـ اس الـ نـ اراات في .(٪ 0.8 u عن تـ سـ فر لـ م الـ ثلاثـ ة TAS جمـ يع في mf الـ حالات كـ ل تـ تـ بع الاتـ صال .(الـ توالـ ي عـ لي ٪ 0.7 و ٪ 0.2 ، ٪ 0.3) الايـ جابـ يه الأطـ فال ايـ جابـ يه حالات.

:الـ نطاق) الـ مائـ ة في 0.7 إيـ جابـ يا معدلا يـ بـ ين ما وهو ، شخـ صا 23 477 مجموعه ما اراخـ تب تـ م ، لـ لمهاجريـ ن الـ صحي الـ فحص خلال ومن 2003 في الـ قططـ بـ ين ايـ فرمكـ كـ تـ ين الـ سـ نوي الـ علاج بـ دا ، نـ اراتـ يوتـ ين محافـ ظه في .2002-2017 سـ نوات مدي عـ لي (٪ 0.1-2.7 ، 2017 ابـ ريـ ل/نـ يـ سان من واعـ تـ بارا .2015 في ٪ 0.8 لـ ىإ 1995 في ٪ 8.0 من الـ قططـ بـ ين و سطـ انـ تـ شار في انـ خـ فاض إلـ ى ادي مما ، صحي مرفـ ق 34 اطار فـ ي ومـ تابـ عـ تهم الـ فـ نطـ يات/الـ لمـ فـ ية الـ وذمه مرضي من 99 مجموعه ما تـ سجـ يل تـ م.

عمـ لـ يات خلال ومن .الـ تغطـ ية معدلات ارتـ فاع مع MDA وا سعه أجريـ ت 2011 إلـ ى 2002 الـ سـ نـ تـ ين مر عـ لي تـ ايـ لانـ د :ا سـ تـ نـ تاجات الـ تـ قـ يـ يم مـ سوحات خلال من وأظهرت الـ فرعـ ية الـ قريـ ة مـ سـ توي عـ لي LF إر سال مـ ناطق بـ تحديـ د قـ امت ، لـ مـ نـ تظمةوا الـ دوريـ ة الـ ر صد وفـ ي .الـ قـ ضاء LF صحة من الـ تحـ قق من مكـ نـ ت الـ تي الإر سال عـ تـ به معدلات دون ، TAS و MDA- سـ توب مـ سوحات-بـ ها الـ خا صة كمشكـ له الـ لمـ فاويـ ة الـ ف يلاريـ ات داء أزالـ ت تـ ايـ لـ ندفـ ي الـ صحة وزارهبـ ان الـ عالـ مـ ية الـ صحة منظمه اعـ ترفـ ت ،2017 سـ بـ تمـ بر/أيـ لول عامه صحـ ية.

Translated from English version into Arabic by Hamza Shaffison, through

中山大學医学院
SUN YAT-SEN UNIVERSITY SCHOOL OF MEDICINE

泰国是如何消除淋巴丝虫病这一公共卫生问题的？

Sunsanee Rojanapanus, Tanaporn Toothong, Patcharida Boondej, Suwich Thammapalo, Naraporn Khuanyoung, Weena Santabutr, Preecha Prempree, Deyer Gopinath and Kapa D. Ramaiah

摘要

引言：在世界卫生组织东南亚区域的 11 个成员国中，有 9 个国家有淋巴丝虫病。本文介绍了自 2001 年泰国启动消除淋巴丝虫病国家计划至 2017 年消除认证期间所采取的强化干预措施。

方法：2001年泰国启动了一项基线流行病学调查，以确定布鲁丝虫病和班氏丝虫病流行区及其流行情况。在11个淋巴丝虫病（LF）流行省份的357个实施单位（IU）使用乙胺嗪（DEC）和阿苯达唑（ALB）进行群体化疗（MDA）。以亚村（sub-village）为实施单位。2006年在11个LF流行省份进行Stop-MDA调查，对6岁以上人群和≤6岁儿童使用免疫层析测试（ICT）来检测班氏丝虫（*Wuchereria bancrofti*）抗原，通过检测微丝蚴（mf）来确定马来丝虫感染。2011年在那拉提瓦省使用ELISA进行Stop-MDA调查。2012–2013年、2015年和2016–2017年在6–7岁年龄组进行传播评估调查（TAS）。在2002–2017年间，通过全国流动人口健康检查使用ICT测试卡对7个省的流动人口进行加强监测。在4个马来丝虫流行省，自1994年开始开展年度调查，检测家猫的LF情况。2001年开展LF慢性病负担调查，将淋巴水肿/象皮病人登记造册。

结果：2002–2006 年，所有 IU 共实施了 5 轮 MDA。由于持续感染，2007–2011 年 Narathiwat 省的 87 个 IU 需要进行额外的年度 MDA。2002–2012 年期间 MDA 年度全国药物覆盖率为 68.0–95.4%。2006 年在 11 个 LF 流行省份进行的 Stop-MDA 调查结果显示，Narathiwat 省 7 个 IU 中检测到 9 个阳性病例，患病率最高为 0.8%（范围：0.1–0.8%）。在 Narathiwa 省的 TAS-1、TAS-2 和 TAS-3 期间，在抗体阳性儿童中检测到低于马来丝虫 mf 的传播阈值率，分别为 0.3%、0.2%和 0.7%。对所有 3 个 TAS 评估调查发现的所有 mf 病例进行接触者追踪，均未发现阳性病例。

针对流动人口的健康检查，共检测了 23 477 人，2002–2017 年间的阳性率为 0.7%（范围：0.1–2.7%）。在那拉提瓦省，于 2003 年开始对猫进行每年伊维菌素治疗，从而使猫的 mf 患病率从 1995 年的 8.0%降至 2015 年的 0.8%。截至 2017 年 4 月，共登记了 99 名淋巴水肿/象皮病患者，并在 34 个卫生机构进行随访。

结论：2002–2011 年间泰国进行了高强度高覆盖率的 MDA。通过定期和常规监测调查，在亚村级别确定了流行区，并通过 Stop-MDA 调查和 TAS 评估调查，证明其已低于达到消除认证的传播阈值。2017 年 9 月，世界卫生组织确认泰国已实现了将淋巴丝虫病作为一个公共卫生问题予以消除。

Translated from English version into Chinese by Xin-Yu Feng, edited by Pin Yang

CHINESE CENTER FOR DISEASE CONTROL AND PREVENTION
NATIONAL INSTITUTE OF PARASITIC DISEASES

Comment la Thaïlande a éliminé le problème de santé publique de la filariose lymphatique

Sunsanee Rojanapanus, Tanaporn Toothong, Patcharida Boondej, Suwich Thammapalo, Naraporn Khuanyoung, Weena Santabutr, Preecha Prempree, Deyer Gopinath et Kapa D. Ramaiah

Résumé

Contexte: La filariose lymphatique est endémique dans neuf des onze pays qui composent la Région Asie du Sud-Est de l’OMS. Le présent article décrit les interventions intensives du Programme national pour l’élimination de la filariose lymphatique en Thaïlande, de son lancement en 2001 à la confirmation de l’élimination en 2017.

Méthodes: Une enquête épidémiologique de référence a été lancée en 2001 pour identifier les zones de filariose brugienne et bancroftienne et tracer les contours des zones d’endémie. Du citrate de diéthylcarbamazine (DEC) et de l’albendazole

(ALB) ont été distribués dans le cadre d'une administration de médicaments en masse (AMM) dans 357 unités de déploiement (UD) au total, dans 11 provinces où la filariose lymphatique était endémique. L'unité de déploiement correspond à un quartier de village. Des enquêtes d'arrêt de l'AMM ont été menées en 2006 dans les 11 provinces d'endémie, sur la population âgée de plus de 6 ans et les enfants de moins de 6, à l'aide d'un test immunochromatographique (TIC) pour rechercher l'antigène de *Wuchereria bancrofti* et de la détection des microfilaires pour détecter *Brugia malayi*. Dans la province de Narathiwat, les enquêtes d'arrêt de l'AMM ont été réalisées en 2011 au moyen d'un test ELISA. Des enquêtes d'évaluation de la transmission ont été menées en 2012-2013, 2015 et 2016-2017 parmi les écoliers de 6-7 ans. La surveillance des populations migrantes au moyen du contrôle sanitaire national des migrants a été intensifiée dans 7 provinces entre 2002 et 2017 et la recherche de l'antigénémie FL au moyen de cartes de test immunochromatographique y a été ajoutée. Dans quatre provinces où *B. malayi* était endémique, des enquêtes annuelles visant à déceler les réservoirs de filariose parmi les chats domestiques ont débuté en 1994. Une étude sur le fardeau de la maladie chronique réalisée pour la filariose lymphatique en 2001 a enregistré le nombre cumulé de personnes présentant un lymphœdème / un éléphantiasis.
Résultats: Au total, cinq cures d'AMM ont été administrées chaque année entre 2002 et 2006 dans toutes les unités de déploiement. Des cures supplémentaires ont été nécessaires dans 87 unités de déploiement de la province de Narathiwat entre 2007et2011, en raison de la persistance de l'infestation. La couverture nationale par l'AMM entre 2002 et 2012 était comprise entre 68,0 % et 95,4 %. Les études d'arrêt de l'AMM de 2006 dans les 11 provinces de FL endémique ont recensé 9 cas positifs pour les microfilaires dans sept unités de déploiement de la province de Narathiwat où la prévalence était maximale à 0,8 % (extrêmes: 0,1-0,8 %). Dans la province de Narathiwat, les études de transmission TAS-1, TAS-2 et TAS-3 ont mesuré des taux de transmission inférieurs au seuil pour les microfilaires de *B. malayi* parmi les enfants ayant des anticorps positifs (0,3 %, 0,2 % et 0,7 % respectivement). Un suivi des contacts de tous les cas positifs aux microfilaires lors des trois études de transmission n'a remonté aucun cas positif.
Le contrôle sanitaire des migrants a permis de tester au total 23 477 personnes, dont 0,7 % étaient positives (fourchette: 0,1–2,7 %), entre 2002 et 2017. Dans la province de Narathiwat, un traitement annuel à l'ivermectine des chats a été mis en place en 2003 et a permis une baisse de la prévalence des microfilaires chez les chats de 8,0 % en 1995 à 0,8 % en 2015. En avril 2017, un total de 99 patients présentant un lymphœdème / un éléphantiasis étaient enregistrés et suivis dans 34 centres sanitaires.
Conclusion: Entre 2002 et 2011, la Thaïlande a procédé à des AMM extensives, avec un fort taux de couverture. Grâce à des enquêtes de suivi périodiques et régulières, les autorités sanitaires ont tracé les zones de transmission de la filariose lymphatique au niveau des quartiers des villages et démontré, par les enquêtes d'évaluation (études d'arrêt de l'AMM et études de transmission), que les taux de transmission étaient inférieurs au seuil, ce qui a permis de confirmer l'élimination de la filariose lymphatique. En septembre 2017, l'Organisation mondiale de la Santé a reconnu que le Ministère de la santé thaïlandais avait éliminé le problème de santé publique de la filariose lymphatique.

Translated from English version into French by Suzanne Assenat, proofread by Gwenaelle Le Jan-Moulart, through

Как Тайланд победил лимфатический филяриатоз как проблему общественного здравоохранения

Сунсани Роджанапанус (Sunsanee Rojanapanus), Танапорн Тутонг (Tanaporn Toothong), Патчарида Бундей (Patcharida Boondej), Сувич Таммапало (Suwich Thammapalo), Нарапорн Хуанйонг (Naraporn Khuanyoung), Вина

Сантабутр (Weena Santabutr), Прича Премпри (Preecha Prempree), Дейер Гопинат (Deyer Gopinath) и Капа Д. Рамайа (Kapa D. Ramaiah)

Реферат

Справочная информация: лимфатический филяриоз эндемичен в 9 из 11 стран-членов ВОЗ Юго-Восточной Азии. В этой статье описывается интенсивное вмешательство в Национальную программу по ликвидации лимфатического филяриоза в Таиланде с момента его появления в 2001 году до подтверждения о его ликвидации в 2017 году.

Методы: В 2001 году началось базовое эпидемиологическое обследование для выявления как филариоза Бэнкрофта, так и бругиоза, а также для определения их эндемичности. Широкомасштабное лечение (ШМЛ) цитратом диэтилкарбамазина (DEC) и альбендазолом (ALB) было осуществлено в общей сложности в 357 пунктах реализации (ПР) в 11 провинциях, в которых эндемичен лимфатический филяриоз (ЛФ). Такими пунктами реализации (ПР) являлись деревни. Обследования в рамках широкомасштабного лечения были проведены в 2006 году в 11 провинциях, для которых эндемичен лимфатический филяриоз,среди населения старше 6 лет и детей до 6 лет с использованием иммунохроматографического исследования (ИХТ) для обнаружения антигена *Wuchereria bancrofti* и выявления микрофилярий (мф) для *Brugia malayi*.В провинции Наратхиват в 2011 году в рамках широкомасштабного лечения проводились исследования с использованием ELISA (ИФА, иммуноферментный твердофазный анализ). Оценочные исследования передачи (ОИП) проводились в 2012–2013, 2015 и 2016–2017 годах среди школьников в возрасте 6–7 лет. В период с 2002 по 2017 г. в семи провинциях проводился более интенсивный эпиднадзор за мигрирующим населением с помощью национального медицинского обследования здоровья мигрантов с использованием тест-карт ИХТ. В четырех провинциях с эндемичным *B. malayi* ежегодные обследования для выявления лимфатического филяриатоза у домашних кошек начались в 1994 году. Обследование бремени хронических заболеваний, проведенное в 2001 году, позволило установить регистр совокупного числа людей с лимфатическим филяриатозом.

Результаты: В течение 2002–2006 годов были проведены пять раундов ШМЛ в год. Дополнительные ежегодные раунды ШМЛ потребовались в 87 ПР провинции Наратхиват с 2007 по –2011 год из-за постоянной инфекции. Годовой национальный охват лекарственными препаратами для ШМЛ за 2002–2012 годы находился в диапазоне от 68,0 % до 95,4 %. Обследования стоп-ШМЛ в 2006 году в 11 провинциях, для которых эндемичен ЛФ, выявили девять случаев положительного анализа на мф в семи ПР в провинции Наратхиват с самой высокой распространенностью 0,8 % (диапазон: 0,1–0,8 %). В процессе ОИП-1, ОИП-2 и ОИП-3 в Наратхиват выявляли уровни ниже уровня передачи для мф *B. malayi* среди антителопозитивных детей (0,3 %, 0,2 % и 0,7 %, соответственно). Отслеживание контактов во всех трех случаях мф во всех трех ОИП не дало положительных результатов.

В ходе проверки здоровья мигрантов было осмотрено в общей сложности 23 477 человек. В результате показатель оказался положительным — 0,7 % (диапазон: 0,1–2,7 %) за 2002–2017 годы. В провинции Наратхиват ежегодное лечение ивермектином среди кошек началось в 2003 году, что привело к снижению распространенности мф у кошек с 8,0 % в 1995 году до 0,8 % в 2015 году. По состоянию на апрель 2017 года в 34 медицинских учреждениях было зарегистрировано 99 пациентов с лимфатическим отеком/элефантиазом, которые находились под наблюдением.

Выводы: Таиланд в период с 2002 по 2011 годы проводил масштабное лечение с большим охватом. Посредством периодических и регулярных мониторинговых обследований очерчивались зоны передачи ЛФ на уровне поселений и демонстрировались в своих оценочных исследованиях — исследованиях стоп-ШМЛ и ОИП — ниже пороговых значений передачи, что позволило провести валидацию устранения ЛФ. В сентябре 2017 года

Всемирная организация здравоохранения признала, что Министерство здравоохранения Таиланда ликвидировало лимфатический филяриатоз как проблему общественного здравоохранения.

Translated from English version into Russian by Michael Kovalevich, proofread by Michael Orlov, through

TRANSLATORS WITHOUT BORDERS

Cómo Tailandia eliminó la filariasis linfática como problema de salud pública

Sunsanee Rojanapanus, Tanaporn Toothong, Patcharida Boondej, Suwich Thammapalo, Naraporn Khuanyoung, Weena Santabutr, Preecha Prempree, Deyer Gopinath y Kapa D. Ramaiah

Resumen

Antecedentes: la filariasis linfática es endémica en nueve de los once Estados Miembros de la Organización Mundial de la Salud en la región del sudeste asiático. El presente artículo describe las intervenciones intensivas del Programa Nacional de Eliminación de la filariasis linfática en Tailandia desde su lanzamiento en 2001 hasta la confirmación de su eliminación en 2017.

Métodos: En 2001 comenzó un estudio de investigación epidemiológico de base para identificar las zonas de filariasis brugiana y bancroftiana y definir su endemicidad. Se implementó la administración masiva de medicamentos (MDA, por sus siglas en inglés) con citrato de dietilcarbamazina (DEC) y albendazol (ALB) en un total de 357 unidades de implementación (UI) en once provincias en las que la filariasis linfática (FL) es endémica. La unidad de implementación (UI) fue una subaldea. En 2006 se realizaron estudios entre la población mayor de 6 años y niños de ≤ 6 años sobre la suspensión de la MDA en las once provincias en las que la FL es endémica utilizando una prueba inmunocromatográgica (ICT) para la detección del antígeno de *Wuchereria bancrofti* y de microfilarias (mf) para *Brugia malayi*. En la provincia de Narathiwat, los estudios sobre la suspensión de la MDA se realizaron en 2011 utilizando ELISA. En 2012–2013, 2015 y 2016–2017 se llevaron a cabo estudios de evaluación de la transmisión (TAS, por sus siglas en inglés) entre alumnos del grupo de edad de 6 a 7 años. Se intensificó la vigilancia de las poblaciones migrantes a través del chequeo médico nacional de los migrantes en siete provincias entre 2002 y 2017 para la antigenemia de FL utilizando tarjetas de ICT. En 1994 se iniciaron estudios anuales para detectar depósitos de FL en gatos domésticos en cuatro provincias en las que el *B. malayi* es endémico. Un estudio de 2001 sobre la carga de una enfermedad crónica para FL estableció un registro del número acumulativo de personas con linfedema / elefantiasis.

Resultados: se realizó un total de cinco rondas de MDA anualmente entre 2002 y 2006 en todas las UI. Se necesitaron rondas anuales adicionales de MDA en 87 UI de la provincia de Narathiwat entre 2007 y 2011 debido a la infección persistente. La cobertura nacional anual de medicamentos con MDA entre 2002 y 2012 fue de entre el 68,0 % y el 95,4 %. Los estudios sobre la suspensión de la MDA en 2006 en las once provincias en las que la FL es endémica encontraron nueve casos positivos de mf en siete UI en la provincia de Narathiwat con la mayor prevalencia siendo del 0,8 % (rango: 0,1–0,8 %). En Narathiwat los TAS-1, TAS-2 y TAS-3 detectaron una transmisión por debajo de los tipos de umbral para mf *B. malayi* entre los niños con anticuerpos positivos (0,3 %, 0,2 % y 0,7 %, respectivamente). La trazabilidad de contactos de todos los casos de mf en los tres TAS no dio ningún caso positivo.

A través del chequeo médico de los migrantes, se examinó un total de 23 477 personas, que mostraron un tipo positivo del 0,7 % (rango: 0,1–2,7 %) entre los años 2002 y 2017. En la provincia de Narathiwat, el tratamiento anual con ivermectina entre los gatos comenzó en 2003, lo que generó una disminución de la prevalencia de mf entre los gatos del 8,0 % en 1995

al 0,8 % en 2015. A partir de abril de 2017, se registró un total de 99 pacientes con linfedema / elefantiasis y se les dio un seguimiento en 34 centros de salud.

Conclusiones: entre los años 2002 y 2011, Tailandia llevó a cabo una amplia MDA con altas tasas de cobertura. Mediante los estudios de seguimiento periódicos y regulares, delimitó las zonas de transmisión de FL a nivel de subaldeas y, a través de sus estudios de evaluación, los estudios sobre la suspensión de la MDA y los TAS, demostró una transmisión por debajo de los tipos de umbral, lo que permitió la confirmación de la eliminación de FL. En septiembre de 2017, la Organización Mundial de la Salud reconoció que el Ministerio de Salud de Tailandia había eliminado la filariasis linfática como problema de salud pública.

Translated from English version into Spanish by Mayra León, proofread by Sandra González, through

TRANSLATORS
WITHOUT BORDERS
